# Ultra-low percolation threshold in ferrite-metal cofired ceramics brings both high permeability and high permittivity

**DOI:** 10.1038/srep07580

**Published:** 2015-01-05

**Authors:** Liang Wang, Yang Bai, Xuefei Lu, Jiang-Li Cao, Li-Jie Qiao

**Affiliations:** 1 Key Laboratory of Environmental Fracture (Ministry of Education), University of Science and Technology Beijing, Beijing 100083, China

## Abstract

High permeability and high permittivity are hard to be achieved simultaneously, either in single-phased materials or in composite materials, such as ferrite-ferroelectric ceramic composites and ferrite-metal percolative composites. In this work, ultra-low percolation threshold is achieved in NiZnCu ferrite-Ag cofired ceramics, which endows the composite with both high permeability and high permittivity by minimizing the negative effect of nonmagnetic conductive fillers on magnetic properties. The percolation threshold is controlled by the temperature matching between ferrite densification and Ag melting. A thin and long percolative net forms between large ferrite grains under a proper cofiring process, which brings a low percolation threshold of 1.21vol%, more than one order of magnitude lower than the theoretical value of 16vol%. Near the ultra-low threshold, the composite exhibits a high permeability of 585 and a high permittivity of 78.

Permittivity and permeability are the two most important features for electromagnetic materials. Recently, the trend in electronics towards small size and high integration promotes the demand for multifunctional electromagnetic materials, such as those with both high permeability and permittivity. Since the desired property cannot be obtained in single-phased materials, people turn to composites, including ferrite-ferroelectric ceramic composites and ceramics-metal percolative composites, but the achievements were still not satisfying. In ferrite-ferroelectric ceramic composites, the permeability and permittivity are determined by the values of each phase and the volume ratio^1^,^2^,^3^,^4^,^5^,^6^. However, the dilution effect prevents producing high permeability and high permittivity simultaneously, although the ferrite phase has high permeability and the ferroelectric phase has high permeability, which follows the effective medium theory. When there is 10 vol% ferroelectric ceramics, the permeability of the composite will be only 1/5 of that of the pure ferrite; and vice versa. For example, Zheng et al. prepared the composites with semiconductive ferrite grains coated by insulating ferroelectric thin films and a ultra-high permittivity of ~75000 was achieved due to a microstructure similar to boundary layer capacitor, while the permeability of 162 is not high enough^5^. Actually, the percolation system is an efficient way to modify the electromagnetic properties nonmonotonically. When the amount of conductive fillers in an insulating matrix approaches a threshold, the composite exhibits a giant permittivity due to the formation of percolative capacitor^6^. This has been well proved in various composite materials, such as polymer-metal composites^7^,^8^,^9^,^10^, polymer-carbon composites^11^,^12^,^13^,^14^ and ceramic-metal composites^6^,^15^,^16^,^17^. However, up to now, the percolation system fails to produce high permeability. In 2005, Shen et al. developed a ferrite-Ni-polymer three-phased composite with electric and magnetic double percolation, and enhanced the permeability by adding Ni in this serial of composites, but the permeability is still low (μ ≈ 30) due to the nonmagnetic polymer matrix^18^. Here, we prepared NiZnCu ferrite-Ag cofired ceramics with an ultra-low percolation threshold of 1.21 vol%, which is one order of magnitude lower than the reported values of ceramic based composites. Because the ultra-low Ag amount minimizes the negative effect of nonmagnetic conductive filler on the magnetic properties of ferrite matrix, the composite exhibits a really high permeability of 585 and a high permittivity of 78 near the ultra-low threshold.

In this work, Ag with melting point of 961°C was used as conductive filler in the composites because of the high conductivity and high resistance to oxidation. Two types of NiZnCu ferrites, Ni_0.2_Zn_0.6_Cu_0.2_Fe_2_O_4_ (NCZ1) and Ni_0.35_Zn_0.6_Cu_0.05_Fe_2_O_4_ (NCZ2), were used as soft magnetic matrix; they had different densification temperatures of 935°C and 1050°C, but similar electromagnetic properties. To control the morphology of Ag filler, the samples were prepared by two-step sintering strategy^19^, where they were heated to a higher temperature T_1_, immediately cooled to a lower temperature T_2_, and then hold for two hours.

## Results and discussions

**Figure 1:**
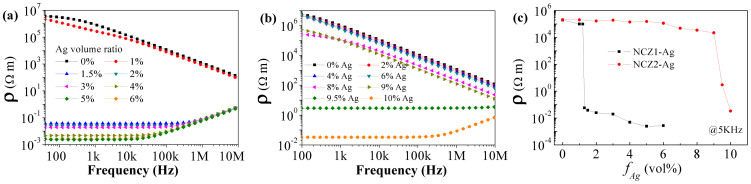
The frequency dependence of electric resistivity of (a) NCZ1-Ag and (b) NCZ2-Ag composites, as well (c) their composition dependence.

**Figure 2:**
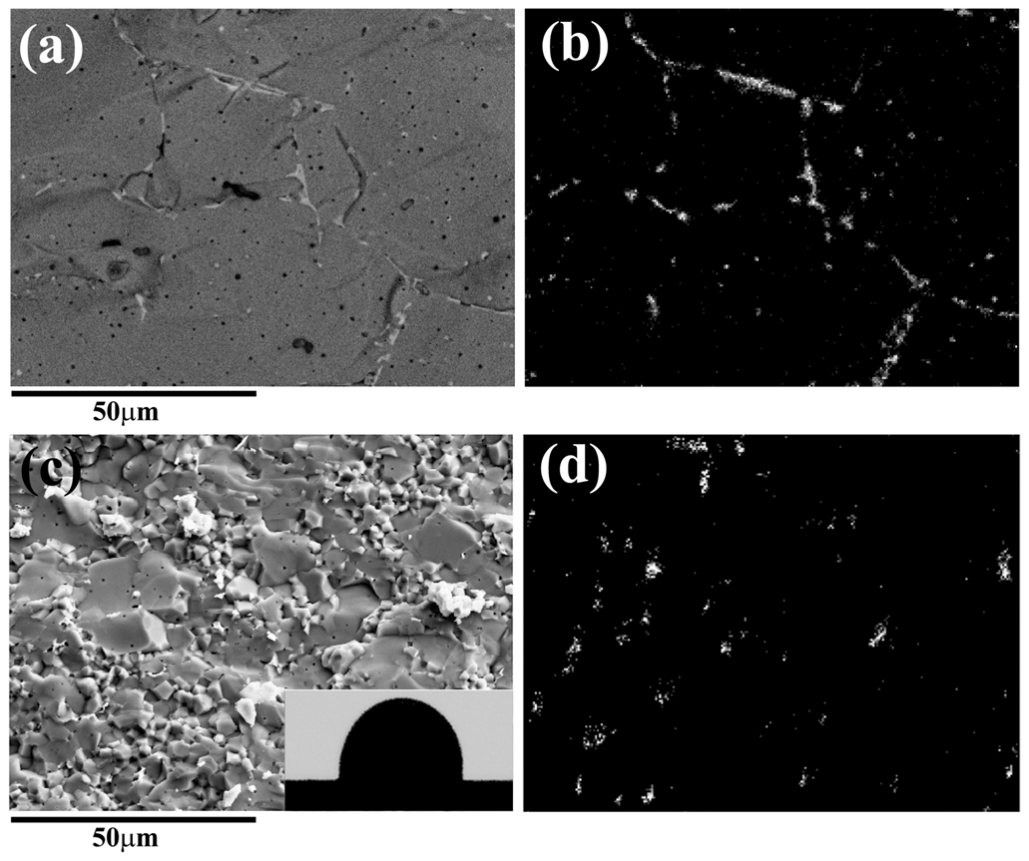
The backscattered electron (a & c) images and energy-dispersive x-ray spectroscopy (b & d) of NZC1-Ag (a & b) and NZC2-Ag composites (c & d) both with 2 vol% Ag. In the backscattered electron images, the white part is silver and the grey part is ferrite. The inset in (c) shows the wetting angle of Ag on ferrite at 970°C.

At the percolation threshold, the conductive fillers connect each other and form a conductive net in the insulating matrix, so that the composite abruptly changes from a dielectric to a conductor. The break of resistivity is clear in the dependence of electric resistivity (Fig. 1), so that the percolation threshold *f_c_* of the samples was characterized and listed in Table 1. In the conductivity measurement, no electrode was pasted on both sides of the sample to confirm the formation of fully percolative net. If electrodes were pasted on both sides of the sample, partial percolation net would also induce a conductive character in the measurement and lead to a false value of percolation threshold. The NCZ2-Ag composite has *f_c_* = 9.5 vol%, which is lower than the theoretical value of 16 vol%, but is similar to Shen's result^18^. On the contrary, the NCZ1-Ag composites have much lower *f_c_*, which originates from the distinctive morphology of Ag in the composites. It originates from the remarkably different Ag morphology. Ag forms a long and thin net homogeneously in the NZC1 ferrite matrix (Fig. 2 (a), (b)); while it is agglomerated to droplets and dispersed among NCZ2 grains (Fig. 2 (c), (d)).

The *f_c_* in a percolation system is not only affected by the volume ratio of conductive fillers but also by the morphology. If there are spherical fillers, the theoretical *f_c_* is ~16 vol%. If the fillers have large aspect ratio, the *f_c_* drops dramatically^20^. The *f_c_* for rodlike fillers with radius of *r* and length of *L* can be expressed as: 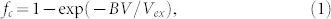
where *B* = 2.7, *V* = *πr*^2^*L* and *V_ex_* = *πrL*^2^ 21. It was reported that the polymer-carbon nanotubes composites had ultra-low low *f_c_* of ~1 vol%^11^,^12^. However, such low *f_c_* has never been reported in ceramic based composite, because carbon nanotubes will burn out in the high temperature sintering process. In this work, the long and thin percolative net is formed in NCZ1-Ag composite under a well controlled cofiring process, and endows it with an ultra-low *f_c_*.

**Figure 3:**
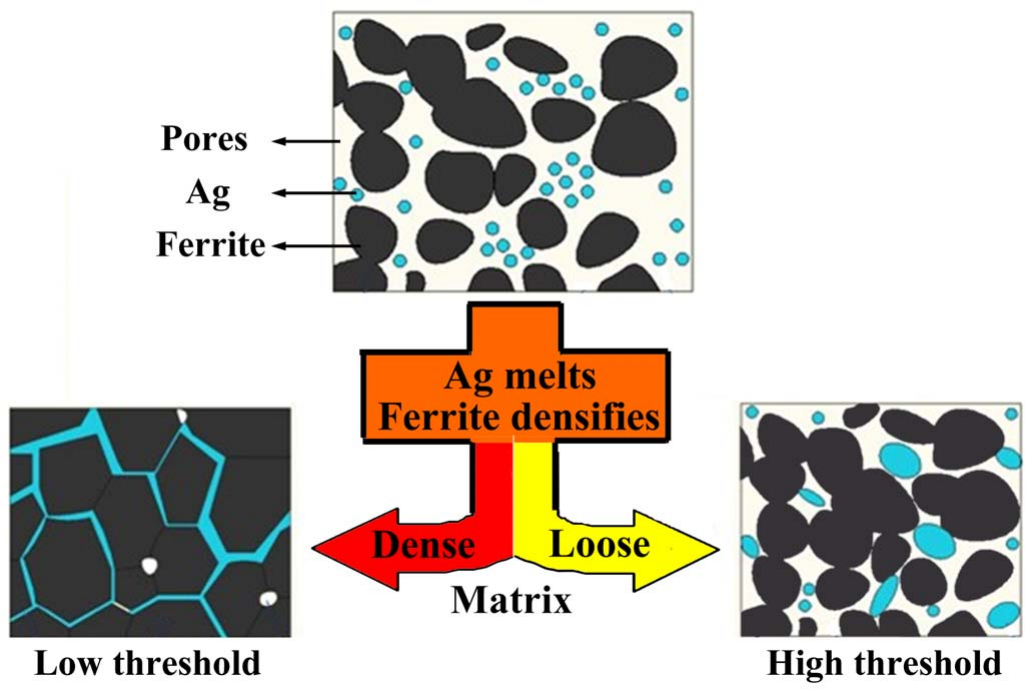
Schematic of Ag melting process in dense or loose matrix.

The *f_c_* is determined by the Ag morphology in composites, which is controlled by the temperature matching between ferrite densification and Ag melting. NZC1 ferrite has largely densified when Ag melts, i.e. grains grow obviously and stack compactly. In a dense microstructure, liquid Ag extends along the narrow grain gaps because of the extruding of large ferrite grains, so that a thin and long percolative net forms easily, as illustrated in Fig. 3. On the contrary, NZC2 ferrite does not begin densifying as Ag melts, whose measured relative density is only 62% at 960°C. In a loose matrix full of pores and gaps, liquid Ag is agglomerated to droplets to minimize the surface energy due to the poor wettability with ferrite (inset in Fig. 2 (c)). Finally, spherical Ag fillers are dispersed among ferrite grains (Fig. 3), which works against forming percolative net.

The sintering process also affects the percolative character by microstructure modification. As shown in Table 1, faster heating rate improves the growth of large grains and helps limiting the Ag particle distribution, so the *f_c_* is lower. If T_1_ ≥ 1150°C or T_2_ ≥ 1035°C, Ag agglomeration occurs to prevent forming percolative net. To achieve ultra-low *f_c_*, T_1_ = 1100°C and T_2_ = 935°C are selected for further investigation.

**Figure 4:**
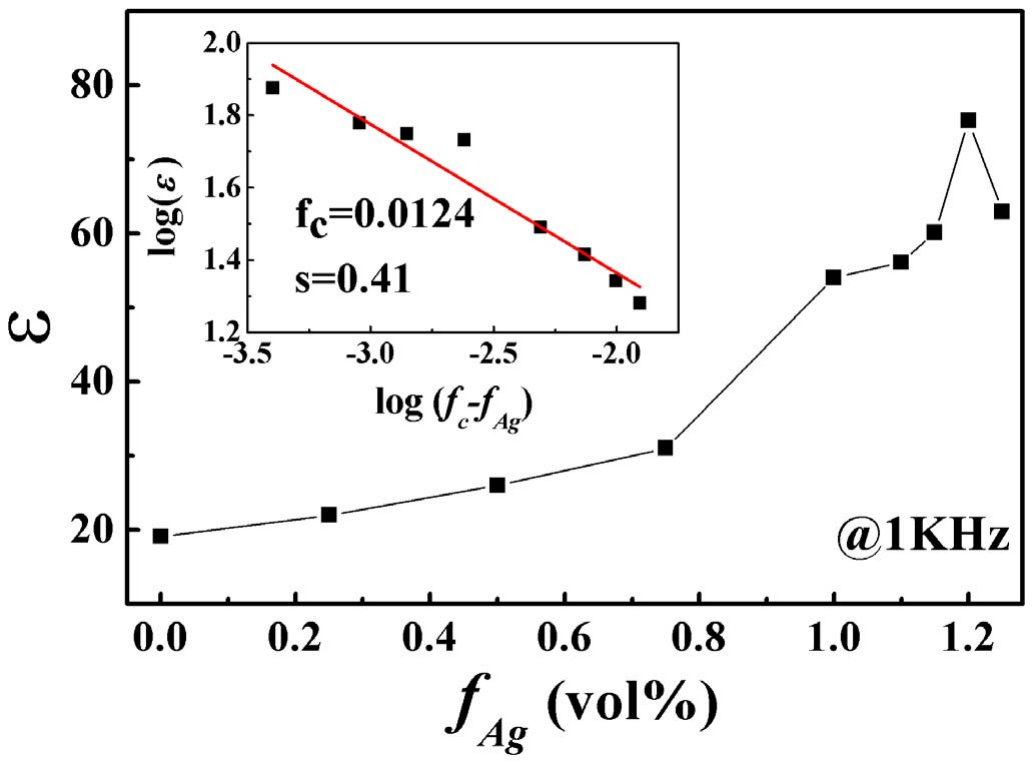
Variation of the permittivity of NZC1-Ag composites with Ag volume ration.

The permittivity of the NCZ1-Ag composites with 0~1.25 vol% Ag is shown in Fig. 4. The permittivity increases rapidly above *f*_Ag_>1.1 vol% and reaches a maximum of 78 at *f*_Ag_ = 1.2 vol%, which is much higher than that of pure ferrite (~19). The variation of permittivity can be characterized by the power law as follow, 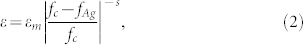
where *ε_m_*is the permittivity of ferrite matrix, *s* is the critical exponent^22^,^23^. Then a precise percolation threshold of *f_c_* = 1.21% and *s* = 0.11 are obtained based on the fitting of experimental data. Such low threshold is similar to some carbon nanotube-polymer composites^11^, but is much lower than the reported values in ceramic based composites^6^,^15^,^16^,^17^.

**Figure 5:**
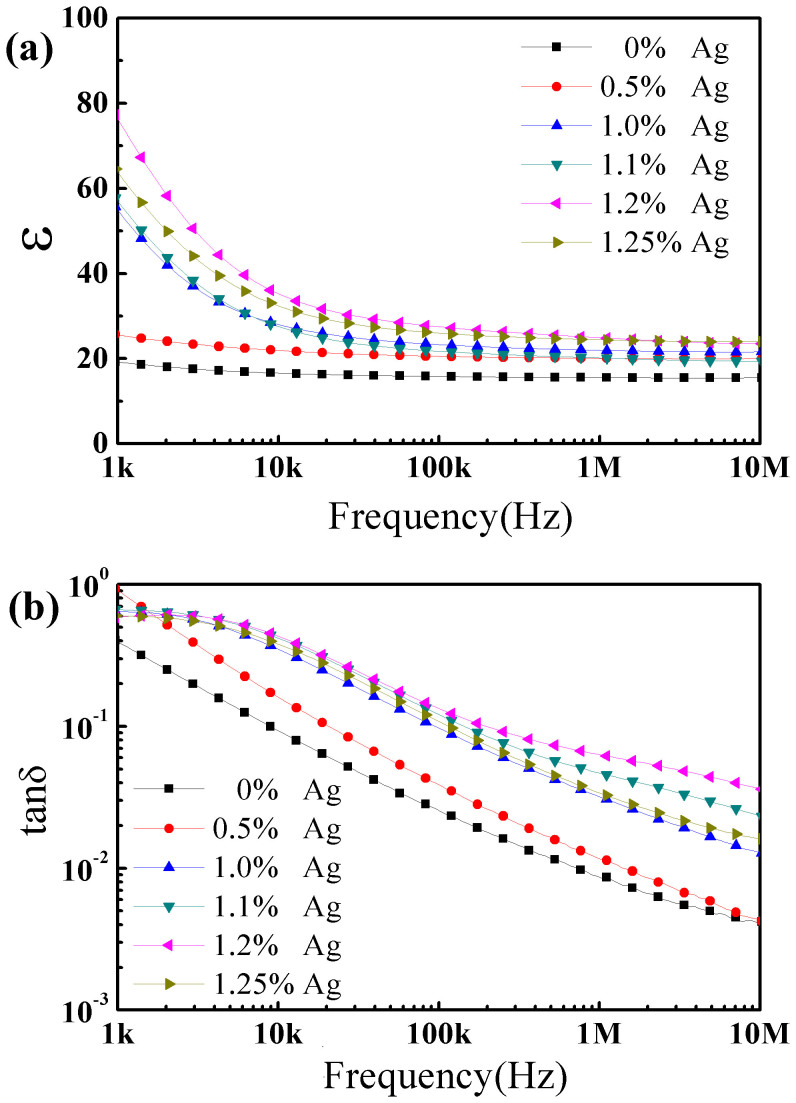
Frequency dependence of (a) permittivity and (b) loss for NCZ1-Ag composites.

The frequency dependence of permittivity and loss tangent is shown in Fig. 5. The permittivity decreases with increasing frequency, following the percolation theory that as *f_Ag_→f_c_*, 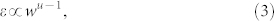
where *w* = 2π*v*, *v* is the frequency, and *u* is a constant. In addition, at the percolation threshold, the dielectric loss is still small. The Maxwell-Wangner effect also contributes for the permittivity in low frequency range.

**Figure 6:**
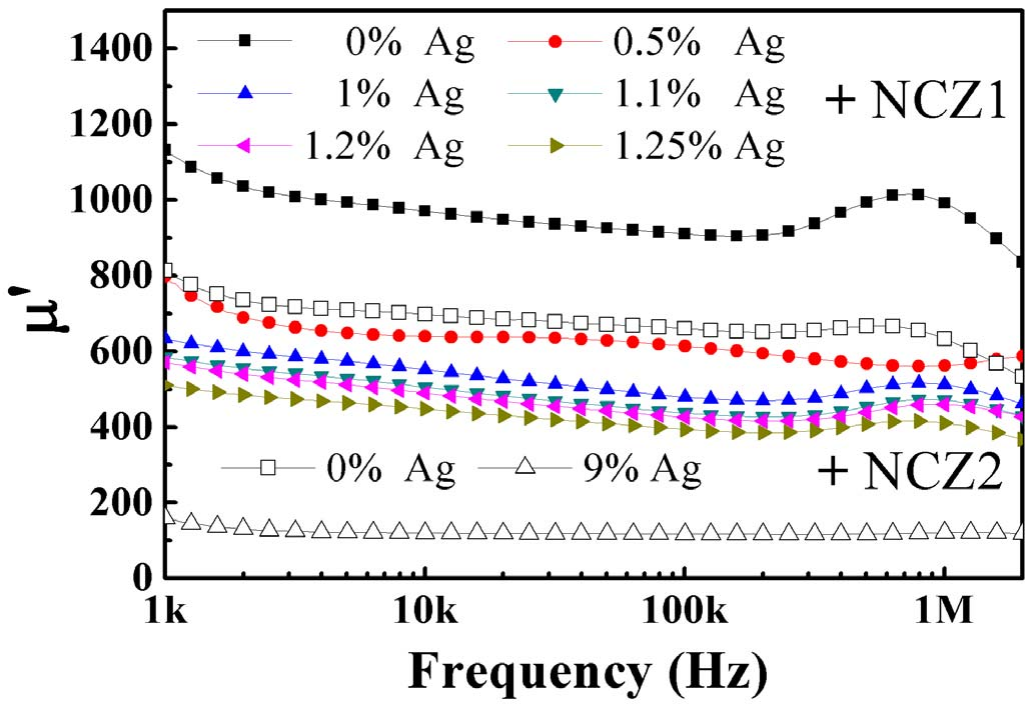
Frequency dependence of permeability for NCZ1-Ag and NCZ2-Ag composites.

Fig. 6 shows the permeability of the NZC1-Ag and NZC2-Ag composites. The permeability decreases with increasing Ag amount. Introducing nonmagnetic Ag in the soft magnetic ferrite matrix will interrupt the magnetic lines of force and produces the demagnetizing field, which can be regarded as an increase of equivalent magnetocrystalline anisotropy. The effects of magnetocrystalline anisotropy *K_1_* and saturation magnetization *M_s_* on the permeability follow^24^, 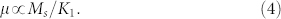
Since the induced field around metal inclusions weakens rapidly with distance, the thin percolation net can minimize the negative effect on magnetic property. The NCZ1-Ag composite has a high permeability of 585 near the *f_c_*, about 55% of pure ferrite's permeability. By contrast, for NCZ2-Ag composite, the permeability drops ~80%, from 735 to 130. It indicates that the ultra-low *f_c_* is efficient to maintain a high permeability in a ferrite-metal composite. That is helpful to produce composites with both high permeability and permittivity.

In summary, the NiZnCu ferrite-Ag cofired ceramics were successfully prepared and its percolation threshold was decreased to an ultra-low value of 1.21 vol%, one order of magnitude lower than the theoretical value, and is much lower than previous reports in ceramic based composites. The *f_c_* is determined by the Ag morphology in composites, which is controlled by the temperature matching between ferrite densification and Ag melting. If Ag melts in a dense matrix, it tends to form a long and thin conductive net, so that the *f_c_* drops to a very low value. The composite exhibits a really high permeability of 585 and a high permittivity of 78 near the *f_c_*. This work provides a way to produce materials with both high permeability and permittivity to promote the development of multifunctional and miniaturizated electronic products. In addition, it founds a simple and effective method to control the percolation threshold in a large range for ceramic based composites, which is helpful to enhance their performance and widen the application.

## Methods

### Preparation of cofired ceramic composite

The Ni_0.4-x_Zn_0.6_Cu_x_Fe_2_O_4_ (where x = 0.05 and 0.2) powders were prepared by the solid reaction method. The analytical grade raw materials of Fe_2_O_3_, NiO, ZnO and CuO were weighted according to the molecular formula and mixed in ball mill for 4 h using a planet mill. After calcined at 760°C for 4 h, the obtained ferrite powders were mixed with spherical silver powder (1 μm, 99.9%) in ball mill. Then, the mixture was granulated using 5% polyvinyl alcohol solution as binder-lubricant. After dry-press, the pellet and toroidal samples were sintered in a two-step sintering strategy.

### Characterization of the samples

The bulk density of the sintered samples was measured using Archimedes' method. The wettability was measured by the high temperature wetting angle measurement instrument (OCA 20LHT-SV). The microstructure of the samples was observed by scanning electron microscope (SEM, JSM-6510A). The permeability, permittvity and conductivity were measured by Agilent 4294A impedance analyzers.

## Author Contributions

Y.B. designed the experiments and analyzed the results. L.W. prepared the samples and characterized the samples. X.L. characterized some of the samples. J.L.C. analyzed some results. L.J.Q. guided the work and analysis. Y.B. wrote the paper.

## Figures and Tables

**Table 1:** The percolation thresholds of the composites

		T_1 _(°C)	T_2 _(°C)	heating rate (°C/min)	*f_c_*
1	NCZ1	1050	935	10	3.5%
2	NCZ1	1100	935	10	1.3%
3	NCZ1	1150	935	10	2%
4	NCZ1	1100	935	1	2.5%
5	NCZ1	1100	935	5	2%
6	NCZ1	1150	1035	10	2%
7	NCZ1	1150	1135	10	2%
8	NCZ2	1200	1050	10	9.5%

## References

[b1] Qi X. *et al.* ferroelectric ferromagnetic composite material with significant permeability and permittivity. Adv. Func. Mater. 14, 920–926 (2004).

[b2] Shen J. H., Bai Y., Zhou J. & Li L. T. Magnetic properties of a novel ceramic ferroelectric-ferromagnetic composite. J. Am. Ceram. Soc. 88, 3440–3443 (2005).

[b3] Bai Y. *et al.* A ferromagnetic ferroelectric cofired ceramic for hyper-frequency. J. Appl. Phys. 101, 083907 (2007).

[b4] Huang J. Q. *et al.* A percolative ferromagnetic-ferroelectric composite with significant dielectric and magnetic properties. Adv. Mater. 19, 437–440 (2007).

[b5] Zheng H. *et al.* Super high threshold percolative ferroelectric/ferromagnetic composite ceramics with outstanding permittivity and initial permeability. Angew. Chem. Int. Ed. 48, 8927–8930 (2009). 10.1002/anie.200904269 19847836

[b6] Pecharromán C. *et al.* New percolative BaTiO_3_-Ni composites with a high and frequency-independent dielectric constant (ε_r_ ≈ 80000). Adv. Mater. 13, 1541–1544 (2001).

[b7] Panda M., Srinivas V. & Thakur A. K. Surface and interfacial effect of filler particle on electrical properties of polyvinylidene fluoride/nickel composites. Appl. Phys. Lett. 93, 242908 (2008).

[b8] Deepa K. S. *et al.* Effect of interparticle distance and interfacial area on the properties of insulator conductor composites. Appl. Phys. Lett. 94, 142902 (2009).

[b9] Huang X., Jiang P. & Xie L. Ferroelectric polymer/silver nanocomposites with high dielectric constant and high thermal conductivity. Appl. Phys. Lett. 95, 242901 (2009).

[b10] Li Y. *et al.* Large dielectric constant and high thermal conductivity in poly(vinylidene fluoride)/barium titanate/silicon carbide three-phase nanocomposites. ACS Appl. Mater. Interfaces 3, 4396–4403 (2011). 22008305 10.1021/am2010459

[b11] Yuan J. K. *et al.* High dielectric permittivity and low percolation threshold in polymer composites based on SiC-carbon nanotubes micro/nano hybrid. Appl. Phys. Lett. 98, 032901 (2011).

[b12] He F., Lau S., Chan H. L. & Fan J. High dielectric permittivity and low percolation threshold in nanocomposites based on poly(vinylidenefluoride) and exfoliated graphite nanoplates. Adv. Mater. 21, 710–715 (2009).

[b13] Dang Z. M. *et al.* Giant dielectric permittivity in functionalized carbon- nanotube/electroactive-polymer nanocomposites, *Adv*. Mater. 19, 852–857 (2007).

[b14] Huang Y. Y. & Terentjev E. M. Tailoring the electrical properties of carbon nanotube-polymer composites. Adv. Funct. Mater. 20, 4062–4068 (2010).

[b15] Wang Z. *et al.* Ag nanoparticle dispersed PbTiO_3_ percolative composite thin film with high permittivity. Appl. Phys. Lett. 93, 222901 (2008).

[b16] George S., James J. & Sebastian M. T. Giant permittivity of a bismuth zinc niobate–silver composite. J. Am. Ceram. Soc. 90, 3522–3528 (2007).

[b17] Shi Z. C. *et al.* Random composites of nickel networks supported by porous alumina toward double negative materials. Adv. Mater. 24, 2349–2352 (2012). 22499287 10.1002/adma.201200157

[b18] Shen Y., Yue Z. X., Li M. & Nan C. W. Enhanced initial permeability and dielectric constant in a double-percolating Ni_0.3_Zn_0.7_Fe_1.95_O_4_-Ni-polymer composite. Adv. Funct. Mater. 15, 1100–1103 (2005).

[b19] Chen I. W. & Wang X. H. Sintering dense nanocrystalline ceramics without final-stage grain growth. Nature 404, 168–171 (2000). 10724165 10.1038/35004548

[b20] Li J. *et al.* Correlations between percolation threshold, dispersion state, and aspect ratio of carbon nanotubes. Adv. Func. Mater. 17, 3207–3215 (2007).

[b21] Nan C. W. Physics of inhomogeneous inorganic materials. Prog. Mater. Sci. 37, 1–116 (1993).

[b22] Dang Z. M., Lin Y. H. & Nan C. W. Novel ferroelectric polymer composites with high dielectric constants. Adv. Mater. 15, 1625–1629 (2003).

[b23] Pecharroman C. & Moya J. S. Experimental evidence of a giant capacitance in insulator-conductor composites at the percolation threshold. Adv. Mater. 12, 294–297 (2000).

[b24] Bai Y., Zhang W. J., Qiao L. J. & Zhou J. The low-fired Y-type hexagonal ferrite for hyper frequency applications. J. Adv. Ceram. 1, 100–109 (2012).

